# The Anti-Apoptotic Bcl-x_L_ Protein, a New Piece in the Puzzle of Cytochrome C Interactome

**DOI:** 10.1371/journal.pone.0018329

**Published:** 2011-04-18

**Authors:** Ivano Bertini, Soizic Chevance, Rebecca Del Conte, Daniela Lalli, Paola Turano

**Affiliations:** 1 Magnetic Resonance Center (CERM), University of Florence, Sesto Fiorentino, Florence, Italy; 2 Department of Chemistry, University of Florence, Sesto Fiorentino, Florence, Italy; University of Naples, Italy

## Abstract

A structural model of the adduct between human cytochrome c and the human anti-apoptotic protein Bcl-x_L_, which defines the protein-protein interaction surface, was obtained from solution NMR chemical shift perturbation data. The atomic level information reveals key intermolecular contacts identifying new potentially druggable areas on cytochrome c and Bcl-x_L_. Involvement of residues on cytochrome c other than those in its complexes with electron transfer partners is apparent. Key differences in the contact area also exist between the Bcl-x_L_ adduct with the Bak peptide and that with cytochrome c. The present model provides insights to the mechanism by which cytochrome c translocated to cytosol can be intercepted, so that the apoptosome is not assembled.

## Introduction

Cytochrome c is a small soluble heme protein loosely associated with the inner membrane of the mitochondrion, where it acts as an electron carrier between the two terminal complexes of the respiration chain, cytochrome bc_1_ and cytochrome c oxidase [1], [2]. The release into the cytosol of cytochrome c is a critical early event in mitochondrially mediated apoptotic cell death [3]. Upon extrusion into the cytosol, cytochrome c forms the apoptosome with Apaf-1 and pro-caspase-9, initiating the caspase cascade of reactions that leads to apoptosis [4]. In absence of cytochrome c, cytosolic Apaf-1 is unable to bind pro-caspase-9 and caspase activation does not occur. Despite its celebrity, the mechanism of cytochrome c release remains largely elusive. It has been proposed to occur in two steps: the upstream event of cytochrome c dissociation from the inner membrane that renders it available for the subsequent release into the cytosol upon permeabilization of the outer mitochondrial by oligomeric pro-apoptotic members of the Bcl-2 family of proteins [5]. Oxidative damage of cardiolipin, a phospholipid that constitutes about 20% of the total lipid composition of the inner membrane, may cause the cytochrome c detachment from the inner membrane [5], [6]. The external membrane permeabilization step is both positively and negatively regulated by members of the Bcl-2 family of proteins [7], [8], [9], through their cytosol-to-external mitochondrial membrane redistribution by means of activated processes [10], [8]. The BH3-only proteins initiate apoptosis through binding to pro-apoptotic Bax or Bak and recruiting them to the membrane, where they form large complexes that generate membrane spanning pores, hence making the membrane permeable [11]. Anti-apoptotic members of the Bcl-2 family, such as Bcl-x_L_, are structurally similar to Bax but inhibit the membrane permeabilization process, do not oligomerize and do not form pores [12]. They might inhibit apoptosis by acting as if they were a dominant-negative version of Bax by competing with it for binding to the outer membrane [12].

Pro-survival proteins like Bcl-x_L_ do prevent cytochrome c release into the cytosol: a number of diverse protein-protein interactions have been proposed to be at the basis of such a process. There have been reports that Bcl-x_L_ can block the formation of the apoptosome associating itself with Apaf-1 and caspase-9 to produce an anti-apoptotic ternary complex [13], [14]. On the other hand cytochrome c was found to interact specifically with Bcl-x_L_ *in vitro* with an affinity that closely matches the reported affinities of BH3 peptides/domains for Bcl-x_L_ [15]. The bimolecular binding rate of Bcl-x_L_ to cytochrome c is also within the range set by dimerization of Bcl-2 family proteins, and by BH3–Bcl-2 protein interactions [15].

In the present study, we report an NMR-derived model structure of human Bcl-x_L_ in complex with human cytochrome c, in its iron(II) form that should represent the relevant redox state for heme iron in the reducing environment of the cytosol. Based on this model, insights into the role of specific amino acids on both partner molecules for the establishment of key interactions are obtained that offer structural basis for the rational design of inhibitors.

## Results and Discussion

Chemical shift changes provide a highly sensitive tool for identifying the residues that play a role in interprotein interactions. NMR chemical shift perturbations of backbone amides in Bcl-x_L_ and reduced cytochrome c reveal that the two proteins form detectable amounts of an adduct. The observed chemical shift variations are small (Fig. S1 and Fig. S2), but increase in a saturable manner upon titration (Fig. S3). The interaction between cytochrome c and Bcl-x_L_ has been reported to be strongly dependent on ionic strength [15]: in 50 mM phosphate buffer, the K_d_ of 1.2 10^−7^ M at 80 mM NaCl increases by nearly 12-fold in the presence of 600 mM NaCl. The relatively high concentrations required for the NMR experiments of these two heavily charged proteins (total charge: −14 for Bcl-x_L_ and +9 for cytochrome c) contribute to the increase of the overall ionic strength of the solution, setting us farther from the optimal conditions for the complex formation. Consistently, the K_d_ values estimated from our chemical shift data (Fig. S3), although measured at 50 mM phosphate buffer and 150 mM NaCl, are of the order of 1 mM. The maximum chemical shift variation here observed for cytochrome c resonances is about ¼ of the maximum value reported for cytochrome c in its interaction with cytochrome b_5_, where a K_d_ of 2 mM was estimated [16]. For the same system, increasing salt concentration was reported to lead to the uniform decrease of the observed chemical shift perturbation values for all affected residues of both proteins [17]. The low affinity of the complex combined with the intrinsic low solubility of Bcl-x_L_ prevented us from achieving protein concentrations in solution higher than 500 µM for the anti-apoptotic protein, that would have provided larger amount of the bound state and therefore larger chemical shift perturbations.

An overall increase in ^15^N transverse relaxation rate values, R_2_, is observed upon titration of Bcl-x_L_ with cytochrome c, which is consistent with an increase in the overall tumbling correlation time upon complex formation [18]. An accurate measure of ^15^N R_2_ in the complex, however, was hampered by the low stability of Bcl-x_L_, caused by local sample heating associated to this type of measurements.

Residues whose chemical shift values are affected by the presence of the partner molecule, when mapped on the proteins' surface, were confined to well defined areas, suggesting the formation of a specific, albeit transient, complex. The restraints derived from the NMR experiments were used as input data for docking calculations for the human cytochrome c−Bcl-x_L_ system with the program HADDOCK [19] and unequivocally define the interface on both proteins.

The obtained ensemble of structural models is constituted by a well defined cluster (Table S1) of 128 conformers with backbone RMSD of 0.8±0.5 Å from the overall lowest energy structure. The dominant contribution to the total interaction energy comes from the electrostatic term. This is consistent with the experimental finding that the interaction affinity is reduced by an increase in ionic strength [15].

**Figure 1:**
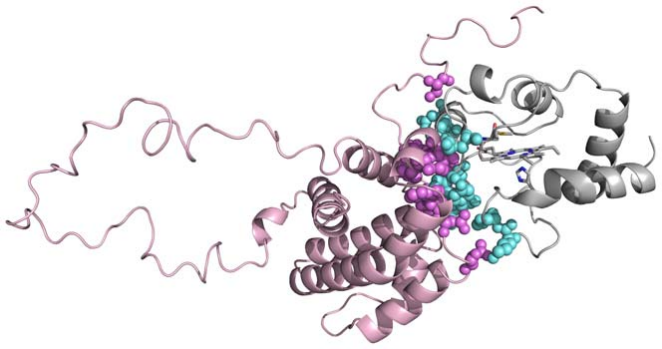
Cytochrome c−Bcl-x_L_ adduct. Residues on cytochrome c (gray and cyan) and Bcl-x_L_ (pink and violet) involved in intermolecular contacts in our structural model. They have been mapped on the lowest energy structure of our cluster of 128 conformers.

A buried surface area of the order of about 2,000 Å^2^ was identified, which contrasts with the short-lived nature of the complex, for which values <1,200 Å^2^ would be expected. A similar situation has been already reported for the cytochrome c−Cu_A_ adduct and explained in terms of a biased picture resulting from the docking procedure [20]; dense networks of intermolecular contacts are provided in the same structural model as if they could be contemporarily present, whereas, reasonably, only a fraction of them is actually formed on average. This situation results from the fact that all the active residues in HADDOCK calculations are treated equivalently, without any attempt to score them on the basis of relative importance to the affinity of the complex. Consistently with this view, the relatively large restraint violation energy hints that none of the calculated structures satisfies all the experimental constraints. Observed chemical shift perturbations in solution reflect the average effect of various interconverting adducts with slightly different binding contacts, as summarized in Table 1. Considering all the identified contacts (as shown in Fig. 1), they define a large and flat contact area between the two partner proteins, that may constitute a valuable guide for future studies aimed at targeting the Bcl-x_L_ - cytochrome c interaction.

**Table 1:** Intermolecular contacts statistics calculated over the 128 model structures of cluster 1 obtained by HADDOCK; all contacts with repetition frequency >30 are listed.

Interacting residues		Interaction type	Frequency
Bcl-x_L_	cytochrome c		
Glu96 (Oε1, Oε2)	Lys53 (Hζ1,Hζ2,Hζ3)	H-bond	141
Arg100 (Hη12, Hη21, Hη22)	Gly41 (CO)	H-bond	64
Tyr101 (Hη)	Ala43 (CO)	H-bond	31
Tyr101 (Cε1,Cγ,Cζ)	Ala43 (Cα,Cβ)	non-bonded contact	195
Asp133 (Oδ2)	Lys25 (Hζ1,Hζ2,Hζ3)	H-bond	87
Asp133 (Oδ1, Oδ2)	His26 (HN)	H-bond	36
Asn136 (Hδ21, Hδ22)	His26 (CO)	H-bond	44
Asn136 (Cβ,Cγ)	Tyr46 (Cδ1)	non-bonded contact	82
Trp137 (Cβ)	Ser47 (Cβ)	non-bonded contact	43
Gly138 (CO)	Gly45 (CO)	non-bonded contact	54
Gly138 (Cα)	Tyr46 (CO,Cα)	non-bonded contact	128
Thr190 (Cγ2)	Lys79 (Cε)	non-bonded contact	42
Phe191 (Cε1)	Ser47 (Cβ)	non-bonded contact	32
Leu194 (CO)	Ala50 (NH)	H-bond	47
Leu194 Backbone (CO)	Ala50 (Cβ)	non-bonded contact	32
Tyr195 (OH)	Lys53 (Hζ1,Hζ2,Hζ3)	H-bond	48
Tyr 195 (Cα,Cδ1)	Ala50 (Cβ)	non-bonded contact	72
Ser203 (Hγ)	Asn54 (Oδ1)	H-bond	45

### Bcl-x_L_ surface contacts with cytochrome c

**Figure 2:**
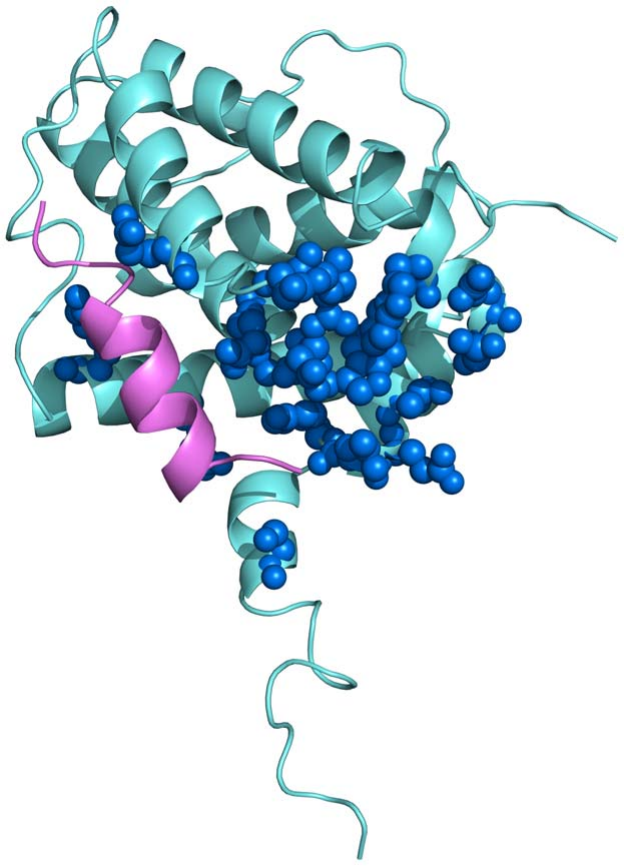
Bcl-x_L_ interaction areas. Ribbon representation of the structure of Bcl-x_L_: the putative transmembrane hydrophobic tail points towards the bottom part of the picture. Residues involved in contacts with cytochrome c are represented as blue spheres. The Bak peptide is shown in magenta and its interaction area has only a few contact points with that defined for cytochrome c.

The structure of Bcl-x_L_ consists of seven helices (according to the PDB analysis of 1LXL) of variable length and a long flexible loop, spanning residues 45 to 84 [21], [22]. The C-terminal part contains a hydrophobic tail proposed to constitute the anchoring point in the membrane bound form. At the base of this short tail the protein fold forms a large and flat surface (Fig. 2), that in the membrane-bound form should be oriented towards the mitochondrion. Residues in contact with cytochrome c are all located in this area. In particular: Glu96 and Tyr101 are on helix-3; Glu129 and Arg139 are the penultimate and the first residue, respectively, of helix-4 and helix-5, which are antiparallel to each other and perpendicular to helix-3; residues 133–138 are on the loop connecting helix-4 to helix-5; Trp181, Glu184 and Asn185 are on helix-7 and Thr190, Glu193 and Leu194 on helix-8, two short helices roughly parallel to helix-3; finally, the last two residues forming contacts are Tyr195, immediately after helix-7 and Ser203 at the base of the C-term tail.

Their spatial location with respect to the anchoring tail suggests that cytochrome c is captured by the protein just at its entrance into the cytosolic space.

Arg139, whose mutation into Glu has been reported to inhibit the anti-apoptotic activity of Bcl-x_L_ [22], is involved in the interaction with cytochrome c and also with the Bak peptide; otherwise the contact surfaces residues of Bcl-x_L_ with the two counterparts do not coincide. Complexation of Bcl-x_L_ with the pro-apoptotic Bak peptide(s) has been reported to occur through an extended interaction with the hydrophobic cleft of Bcl-x_L_ defined by helices 3 and 4; in addition a few charged side chains of opposite signs on the two partners are facing each other [22].

The non-coincidence of the contact surface areas in the two adducts may provide hints for differently targeting the pro-apoptotic and the anti-apoptotic protein-protein interactions.

### Cytochrome c surface contacts with Bcl-x_L_ and comparison with cytochrome c electron transfer complexes

**Figure 3:**
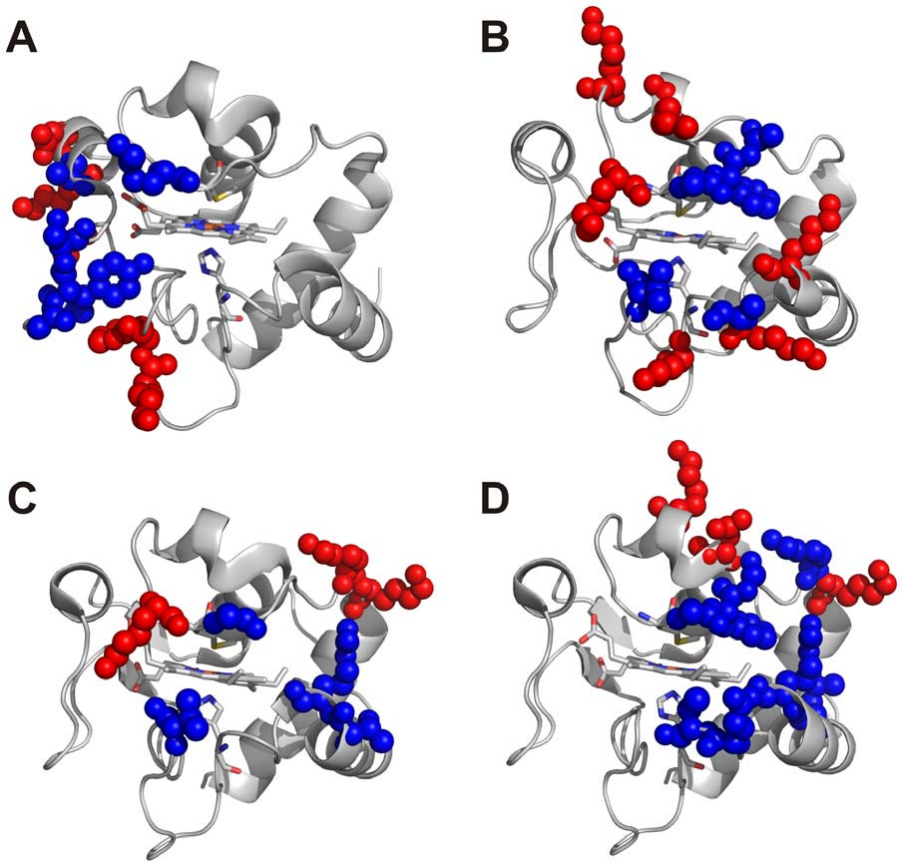
Hydrophobic and electrostatic contacts in cytochrome c complexes. Residues involved in hydrophobic (blue spheres) and in electrostatic/H-bond (red spheres) interactions are shown for: (A) human cytochrome c and Bcl-x_L_, (B) cytochrome c_552_ and cytochrome c oxidase, (C) *S. cerevisiae* cytochrome c and cytochrome bc_1_, (D) *S. cerevisiae* cytochrome c and cytochrome c peroxidase adducts. In the four panels, cytochrome c is represented with an orientation where the “loop face” points towards the observer.

The cytochrome c fold presents five α-helices and a short antiparallel β-strand on one face and two extended loops on the other (Fig. 3) [23], [24], [25], [2]. The two loops sandwich on the heme providing the two axial ligands of the heme iron i.e. His18 and Met80. The porphyrin ring is partially solvent exposed on the side defined by the two loops. Residues on cytochrome c involved in contacts with Bcl-x_L_ are located on the two loops, the helix-3 (also called 50's helix) and on the β-strand (Fig. 3A). Although input active residues in HADDOCK calculations are treated equivalently without any attempt to score them on the basis of relative chemical shit perturbation, the interaction areas resulting from the calculations are centered on the most affected residues i.e., His26 and Gly41. Interestingly, the only known pro-apoptotic mutant of cytochrome is G41S [26], a variant bearing a mutation on a residue of the β-strand found to form an H-bond with Arg100 of Bcl-x_L_ in 64 out of 128 structures of our ensemble. The chemical shift of the amide of Gly41 is the second most affected signal of cytochrome c. However, residues proposed to play a role in the interaction with Apaf-1 [27], [28], with the exception of Lys25, do not match those identified here as contacts with Bcl-x_L_. Lys25 side chain forms an H-bond with Asp133 of Bcl-x_L_ in 87 out of 128 conformers of our cluster 1. Consistently, the two residues adjacent to it, Gly24 and His26, do experience chemical shift perturbations upon binding to Bcl-x_L_, with His26 being the most affected amide on cytochrome c. Unfortunately, the low resolution of the recent structure of the apoptosome obtained by cryo-EM prevented any inference regarding intermolecular contacts involving cytochrome c [29], [30].

The observed distribution of contact residues on cytochrome c differs from that in its electron transfer complexes (Fig. 3B–D), as detailed below.

Efficient electron transfer between cytochrome c and its counterparts in the respiratory chain requires rapid adduct formation and rapid product dissociation as well as the achievement of proper orientation of the partner proteins in the transient adduct to optimize the electron transfer rate. Such requirements are reflected in the high K_d_ values, namely in the µM-mM range [31], [32], and in the nature of key interactions involving surface residues surrounding the heme crevice of cytochrome c. The optimal interfacial arrangement is tuned by hydrophobic interactions among short range contacts. The transient nature of the complex is assured by the possibility to switch on and off the potential electrostatic interactions among residues of different sign surrounding the contact central region on the two proteins. Long-range recognition of the partners is driven by non-specific electrostatic interactions that rely on the presence of large patches of opposite charge on the two protein surfaces.

No structural information is available for the eukaryotic cytochrome c - cytochrome c oxidase complex. Given the high homology in the involved protein domains, the bacterial complex has been proposed in the literature as a suitable model system to achieve functional information that can be extrapolated to its eukaryotic counterpart [20], in spite of the much higher structural complexity of the cytochrome c oxidase of the latter. Even if the per residue contacts may be different in the eukaryotic complex, the overall interaction areas are expected to be the same. In the various conformers of the structural model of the adduct between cytochrome c_552_ and the CuA subunit of cytochrome c oxidase from *Paracoccus denitrificans* (Fig. 3B) [20] common hydrophobic patches (involving residues Ala16, Val26, Ala79, Phe80, and Ala81 on the cytochrome c_552_) are found, while different networks of electrostatic intermolecular interactions are established within negatively charged Asp and Glu contiguous to the central hydrophobic surface area on cytochrome c oxidase and the positively charged Lys residues, namely Lys13, Lys15, Lys19, Lys70, Lys74 and Lys77, surrounding the heme crevice on the cytochrome c_552_.

In the crystal structure of the complex between cytochrome c and cytochrome bc_1_ from *Saccharomyces cerevisiae* (Fig. 3C) [33] the interaction with the subunit cytochrome c_1_ of the enzyme is mainly mediated by non polar contacts involving residues Thr12, Arg13, Val28 and Ala81 on cytochrome c. Weak, polar interactions involving Lys79 and Lys86 are present, while additional electrostatic interactions (i.e. cytochrome c Lys87) have been proposed to modulate intermediate states and the unbinding step.

The crystal structure of the complex between *S. cerevisiae* cytochrome c and its redox soluble partner cytochrome c peroxidase reveals that hydrophobic interactions are the predominant forces holding the complex together (Fig. 3D) [34]. On the side of cytochrome c, they involve residues Leu9, Arg13, Gln16, Cys17, Ala81, Phe82, Gly83 and Lys86. The side chains of Asn70, Lys73 and Lys87 are potentially involved in hydrogen bonds and/or salt bridges.

The key residues for the interaction of cytochrome c with its various redox partners do not coincide but identify similar contact areas. In this binding mode, defined as the “pyrrole II” mode [35], on the side of cytochrome c the interaction is centred on the heme crevice defined by the two loops, where atoms of the porphyrin ring become partially exposed. The differences in the various complexes might account for the structural features of the two examined cytochromes (for example cytochrome c_552_ has a different conformation of the distal loop due to the different length of this structural element: 13 amino acids vs. 17 amino acids in the yeast protein) as well as differences in the nature of residues on the surface the partner proteins. Slightly different slides of the various redox enzymes on cytochrome c surface to optimize intermolecular contacts finely tune the interaction and results in a different involvement of peripheral residues.

Cytochrome c in the anti-apoptotic complex shares only a few contact residues with the electron transfer adducts. Here, the core interactions are centred on the left side (according to the view of Fig. 3) of the heme crevice. The loops are always involved, reflecting the need of conformational adaptability to facilitate an induced fit. At the same time, as the anti-apoptotic interaction with Bcl-x_L_ does not require any electron transfer, doesn't need the involvement of the solvent exposed heme edge.

### Further considerations about the recognition process

Our structural model clearly emerges from the NMR data and is consistent with pro-apoptotic mutations reported for both cytochrome c and Bcl-x_L_. One could question about the relevance of such a weak complex for blocking the apoptosome formation. Nevertheless, two key aspects should be considered. The relatively high ionic strength of the solution needed for *in vitro* experiments (as previously discussed) is such that affinity measurements are done far from the optimal conditions for the interaction, and the resulting K_d_ values are higher than they should be. Another difference between the *in vitro* experiment and the environment inside the cell is the reduced accessible surface area for a membrane tail-anchored protein. In considering this aspect, one should take into account the fact that, in our *in vitro* NMR experiments, both cytochrome c and Bcl-x_L_ can freely diffuse in three dimensions. The *in vivo* anti-apoptotic process of sequestration of cytochrome c by Bcl-x_L_ can be seen as a bait and prey process, where Bcl-x_L_ acts as bait when anchored to the external mitochondrial membrane and therefore has restricted motions and increased local concentration. The prey, cytochrome c, is “fished” for by Bcl-x_L_ once released in the cytosol, where in principle it may be a three dimensional diffusant, but the proximity of the mitochondrial membrane may still influence its diffusion modes. Reducing the dimensionality of the recognition process between the two proteins may lead to a sensible increase in binding efficiency.

### Prospects

Apoptosis normally eliminates cells with damaged DNA or aberrant cell cycle. Pro-survival proteins are therefore potentially oncogenic. Clarifying how the Bcl-2 family governs apoptosis might provide the ability to control the apoptotic threshold.

Conventional cytotoxic therapy indirectly induces apoptosis, but more effective outcomes could be achieved by direct activation of the apoptotic machinery. Promising approaches include impairing expression of pro-survival proteins or identifying drugs that inhibits their action. The identification of interfaces between partner molecules provides targets for pharmacological intervention; the protein-protein interaction surface between Bcl-x_L_ and cytochrome c here identified may offer one of these targets.

## Materials and Methods

### Protein samples

Full length human cytochrome c was expressed and purified as reported in the literature [36] in the unlabeled and ^15^N-labeled form.

The Bcl-x_L_ construct used in our experiments contains residues 1–209 and lacks the C-terminal hydrophobic tail. The construct also has four additional N-term residues (numbers −3 to 0). Unlabeled, ^15^N-labeled and ^13^C,^15^N labeled forms of the protein were used for different NMR experiments. The protein was expressed and purified by ProtEra through a custom protein production service.

Typical protein concentrations for NMR experiments were in the 50 µM to 5 mM range, in 50 mM sodium phosphate buffer at pH 7.3, 150 mM NaCl, 1mM DTT and with 10% D_2_O for lock.

### NMR spectroscopy

All NMR spectra were acquired at 300 K using Bruker Advance spectrometers operating at proton frequencies of 500, 700, 800 and 900 MHz, all equipped with cryoprobes. A table summarizing the NMR experiments performed is given in the Supplemental Material (Table S2). NMR spectra were processed with Topspin version 2.0 and analyzed with the program Cara [37].

#### Interaction studies

Titrations of ^15^N human cytochrome c with unlabeled Bcl-x_L_ and titrations of ^15^N-Bcl-x_L_ with unlabeled cytochrome c were followed through ^1^H-^15^N HSQC. Looking at the ^15^N-enriched Bcl-x_L_ the system was studied until a ratio of Bcl-x_L_∶ cytochrome c 1∶10. Looking at the ^15^N-enriched cytochrome c we could reach a cytochrome c ∶ Bcl-x_L_ ratio of 1∶20.

#### Assignment of Bcl-x_L_

Backbone resonance assignments of Bcl-x_L_ were performed through conventional multidimensional NMR techniques based on triple resonance experiments, as summarized in Table S2. The assignment was carried out starting from the reported assignment (BMRB entry 6578) [38], that refers to a dimeric form of the protein lacking the 45–84 flexible loop. We have accomplished 84% and 80% assignment of the Cα and HN backbone resonances, respectively.

#### R_2_ measurements

The generalized increase in ^15^N R_2_ relaxation rates of Bcl-x_L_ was used to monitor the increase in average molecular size in the presence of 2-fold and 4-fold excess of cytochrome c. The experimental details are provided in Table S2. The local overheating typical of R_2_ measurements affects the stability of Bcl-x_L_, as revealed by ^1^H-^15^N HSQC experiments recorded in an interleaved manner during R_2_ experiments. The effect becomes more important in the presence of cytochrome c and is proportional to its concentration. Nevertheless an overall increase in R_2_, consistent with an increase in the correlation time for tumbling was observed.

### Chemical shift mapping

The interaction between cytochrome c and Bcl-x_L_ was monitored through chemical shift changes of the signals from the backbone amide moieties, whose magnitude increased upon increasing concentration of the titrant (Fig. S1 and Fig. S2). The extent of the changes was quantified through the following equation (Garrett value) [39]:
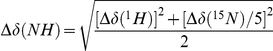
K_d_ values were obtained by plotting the weighted average chemical shift variations of perturbed residues on the ^15^N-enriched Bcl-x_L_ as a function of the concentration of the unlabeled partner cytochrome c (Fig. S3) and were found to be in the 1–3 mM range.

### Model structure calculations

A structural model of the cytochrome c−Bcl-x_L_ adduct was obtained using HADDOCK program [19]. HADDOCK calculations were started with the coordinates of human cytochrome c (PDB id: 1J3S) and human Bcl-x_L_ (PDB id: 1LXL). The starting structure for cytochrome c is actually the first member of an ensemble of 20 NMR conformers.

The docking process in HADDOCK is driven by ambiguous interaction restraints (AIRs), which are derived from the available experimental information on the residues involved in the intermolecular interaction. A distinction is made between active and passive residues: the former are residues which are involved in the interaction and have a high solvent accessibility (i.e. >50%) in the free-form protein, while the latter correspond to solvent-accessible surface neighbors of the active residues.

The active residues defined for the present calculation are listed in Table S3. The solvent accessibility was calculated with the program NACCESS. The HADDOCK docking protocol consisted in three steps: randomization of orientations and rigid body minimization, semi-flexible simulated annealing in torsion angle space and flexible solvent refinement where the structures obtained after the semi-flexible simulated annealing are refined in an explicit solvent layer.

Finally, the solutions were clustered following the two standard criteria of the HADDOCK program i.e., a group of structures forms a cluster if constituted by at least four members having a ligand interface RMSD within 7.5 Å. In our case, these criteria led to the identification of four clusters, that were ranked according to their HADDOCK score (defined as the weighted sum of van der Waals, electrostatic, desolvation and restrains violation energy terms). These four clusters contain 128, 21, 6, and 11 structures, respectively. The structural statistics calculated over all structures of each cluster are shown in Table S1. Cluster 1 is by far the best in terms of RMSD and energy values. The RMSD value reported in the 6^th^ column of Table S1 is the average RMSD from the cluster to the lowest overall energy model, i.e. the lowest energy structure of cluster 1. Cluster 1, with an overall RMSD value of 0.8 Å, unequivocally defines the docking face for both proteins and their relative orientations. Cluster 2 differs from cluster 1 in the relative orientation of the two proteins, although the overall contact surface area is the same.

## Supporting Information

Figure S1 Plot of the chemical shift variation (Garrett values) of the backbone cytochrome c amide signals in the cytochrome c−Bcl-x_L_ adduct. The horizontal line indicates the selected chemical shift perturbation threshold.
(TIF)

Figure S2 Plot of the chemical shift variation (Garrett values) of the backbone Bcl-x_L_ amide signals in the cytochrome c−Bcl-x_L_ adduct. The horizontal line indicates the selected chemical shift perturbation threshold.
(TIF)

Figure S3 Fitting of the weighted average chemical shift variations of three perturbed residues (Leu90, Gly94, Gly200) of the ^15^N-enriched Bcl-x_L_ as a function of the concentration of the unlabeled cytochrome c.
(TIF)

Table S1 Structural statistics calculated over all structures for the 4 clusters obtained by HADDOCK calculations.
(PDF)

Table S2 Acquisition parameters for the NMR experiments; all spectra were acquired at 300 K.
(PDF)

Table S3 HADDOCK active residues for Bcl-x_L_ and cytochrome c.
(PDF)
