# Supplementary material for: The Anti-Apoptotic Bcl-xL Protein, a New Piece in the Puzzle of Cytochrome C Interactome
Source: PLoS One. 2011 Apr 18;6(4):e18329. doi: 10.1371/journal.pone.0018329 (PMC3080137; doi:10.1371/journal.pone.0018329)
Supplement: Table S1 — Structural statistics calculated over all structures for the 4 clusters obtained by HADDOCK calculations. (PDF) [file pone.0018329.s004.pdf]

Table S1. Structural statistics calculated over all structures for the 4 clusters obtained by HADDOCK calculations.

Number of structures within the cluster	van der Waals energy (kcal/mol)	Electrostatic energy (kcal/mol)	Desolvation energy (kcal/mol)	Restrains violations	Average RMSD from the cluster to the lowest overall energy model (Å)	Average RMSD from the cluster to the lowest energy model within the cluster (Å)	Buried surface area ($Å^2$)
128	-59.6±8.4	-535.9±27.6	39.2±3.3	194.7±58.84	0.8±0.5	0.8±0.5	2040.1±81.5
21	-39.5±5.7	-371.2±43.7	39.2±6.8	141.5±48.88	11.5±0.1	1.3±0.8	1599.6±121.6
6	-40.2±5.3	-371.7±31.8	45.0±10.4	116.7±44.73	11.8±0.1	0.8±0.5	1458.1±98.2
11	-43.7±6.0	-291.4±34.4	40.7±10.0	114.2±52.48	8.9±0.2	2.1±1.4	1285.5±40.7
