# Supplementary material for: The Anti-Apoptotic Bcl-xL Protein, a New Piece in the Puzzle of Cytochrome C Interactome
Source: PLoS One. 2011 Apr 18;6(4):e18329. doi: 10.1371/journal.pone.0018329 (PMC3080137; doi:10.1371/journal.pone.0018329)
Supplement: Table S2 — Acquisition parameters for the NMR experiments; all spectra were acquired at 300 K. (PDF) [file pone.0018329.s005.pdf]

Table S2. Acquisition parameters for the NMR experiments; all spectra were acquired at 300 K.

Experiments	Ref.	Dimension of acquired data (nucleus)			Spectral width (ppm)		
		t_1	t_2	t_3	F_1	F_2	F_3
^{1}H-^{15}N-HSQC[a]	[1]	200 or 256 (^{15}N)	1024(^{1}H)	-	13.0	40.0	-
HNCA[b]	[2]	200(^{13}C)	40(^{15}N)	1024(^{1}H)	32.0	36.0	14.0
HN(CO)CA[b]	[2]	200(^{13}C)	40(^{15}N)	1024(^{1}H)	32.0	36.0	14.0
^{15}N R_2[c]	[3]	200(^{15}N)	2048(^{1}H)		32	14	

a Spectra acquired at 500, 800 or 900 MHz. All the spectrometers are equipped with cryo-probe (TXI 5-mm)
b Data acquired using a 700 MHz spectrometer equipped with a cryo-probe (TXI 5-mm)
c Data acquired using a 500 MHz spectrometer equipped with a cryo-probe (TXI 5-mm)

References

1. Sklenar V, Piotto M, Leppik R, Saudek V (1993) Gradient-tailored water suppression for ^{1}H-^{15}N HSQC experiments optimized to retain full sensitivity. Journal of Magnetic Resonance Series A 102: 241-245.
2. Grzesiek S, Bax A (1992) Improved 3D Triple-Resonance NMR Techniques Applied to a 31 KDa Protein. J.Magn.Reson. 96: 432-440.
3. Kay LE, Nicholson LK, Delaglio F, Bax A, Torchia DA (1992) Pulse sequences for removal of the effects of cross correlation between dipolar and chemical-shift anisotropy relaxation mechanisms on the measurement of heteronuclear T_1 and T_2 values in proteins. J.Magn.Reson. 97: 359-375.
