# Supplementary material for: The Anti-Apoptotic Bcl-xL Protein, a New Piece in the Puzzle of Cytochrome C Interactome
Source: PLoS One. 2011 Apr 18;6(4):e18329. doi: 10.1371/journal.pone.0018329 (PMC3080137; doi:10.1371/journal.pone.0018329)
Supplement: Table S3 — HADDOCK active residues for Bcl-xL and cytochrome c. (PDF) [file pone.0018329.s006.pdf]

Table S3. HADDOCK active residues for Bcl-x_L and cytochrome c.

Bcl-x_L	cytochrome c
Q88, E129, D133, T190, F191[a], G196	Q16, V20, G24, H26, G29, G41, A50, K79, M80, V83

a The residue was considered active although it does not result solvent accessible from NACCESS calculations based on the solution structure 1LXL.
